# Testing Crack Resistance of Non-Load-Bearing Ceramic Walls with Door Openings

**DOI:** 10.3390/ma14061379

**Published:** 2021-03-12

**Authors:** Tomasz Kania, Valery Derkach, Rafał Nowak

**Affiliations:** 1 Faculty of Civil Engineering, Wrocław University of Science and Technology, Wybrzeże Wyspiańskiego 27, 50-370 Wrocław, Poland; 2 Research Enterprise for Construction “Institute BelNIIS”, 15 “B”, F. Skoriny str., 220076 Minsk, Belarus; institute@belniis.by; 3 Faculty of Civil and Environmental Engineering, West Pomeranian University of Technology, 70-311 Szczecin, Poland; rnowak@zut.edu.pl

**Keywords:** partition walls, brick walls, bending strength, cracking

## Abstract

Cracking in non-load-bearing internal partition walls is a serious problem that frequently occurs in new buildings within the short term after putting them into service or even before completion of construction. Sometimes, it is so considerable that it cannot be accepted by the occupiers. The article presents tests of cracking in ceramic walls with a door opening connected in a rigid and flexible way along vertical edges. The first analyzes were conducted using the finite element method (FEM), and afterward, the measurements of deformations and stresses in walls on deflecting floors were performed on a full scale in the actual building structure. The measurements enabled to determine floor deformations leading to cracking of walls and to establish a dependency between the values of tensile stresses within the area of the door opening corners and their location along the length of walls and type of vertical connection with the structure.

## 1. Introduction

**Figure 1:**
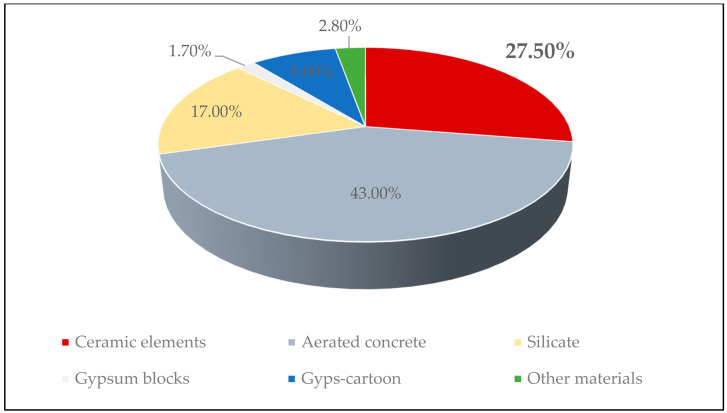
Distribution of the use of wall materials for the construction of non-load-bearing walls in residential buildings in Poland in 2020.

Cracking of partition walls in buildings is a frequent phenomenon. According to the statistical data available in the literature [1,2,3], the displacement of their supporting elements is responsible for 60–70% of damage to walls in Central and Eastern Europe. It results partially from the lack of possibility to analyze accurately spatial relocations of building structures. Application of oversized elements of load-bearing structures in order to limit the cracking of partition walls is an action unjustified economically. Small cracks of filling elements in the building generally are not the causes for concern; however, the commonness of this type of defects, their scale, and often considerable width of cracks are the reasons for expedited repair work, which is often completed still before the structures are handed over for use. The scale of this phenomenon leads to the question about the possibility and methods of counteracting it, the validity of research conducted within this scope, and consistency of requirements both with respect to acceptable deformations of structures supporting partition walls and methods of completing properly their circumferential connections and elements having an effect on cooperation with the building structure. In the countries of Central and Eastern Europe, in the residential buildings, non-load-bearing walls are being made mainly with the use of masonry elements. The percentage of individual building materials for the construction of this type of wall in Poland is shown in Figure 1 [3].

Despite the growing popularity of the gypsum plasterboard lightweight walls in the countries of Central and Eastern Europe, this technology is currently used primarily to make partitions in office and service buildings. In residential buildings constructed in 2020 in Poland, over 95% of non-load-bearing walls were made in masonry technology. Moreover, 27.5% of walls were made of ceramic elements, which indicates the essence of the problem of their cracking undertaken in the presented research.

There have been studies concerning the influence of foundation deformations on the cracking of ceramic walls. It can be concluded from the first research analyses concerning the operation of masonry walls on flexible supports that in order to protect them from cracking, the ratio of deflection of the supporting structure to its span should not exceed 1/2000, and bending tensile strength of the wall should not be lower than 0.21 MPa [4]. In his study [5], Beranek recommends that the bending tensile strength of the walls should be a minimum of 0.1–0.3 MPa. The study [6] presents results of tests concerning the strength of walls with and without window and door openings. The models were made at a scale of 1:3 versus actual dimensions of walls, using appropriately reduced ceramic wall elements. The models were laid on double-span reinforced concrete beams, and they were tested in two stages. In the first stage, a uniformly distributed load was applied using a spreader beam to the top edge of the wall. Then, relocation of the reinforced concrete beam that was a support of the wall in the middle of its span was forced. In the model without openings, the first crack appeared when the ratio of the beam deflection to its span was 1/1000. In the models with openings, cracks were observed slightly later when the ratio of deflection to span was 1/947. Openings in the wall had a significant effect on the layout of cracks. The study [7] presents results of tests and analyses of walls made of full ceramic bricks. It was concluded that admissible deflection of the supporting structure depends on whether it is made with openings or without them. In the case of walls without openings, it was recommended that deflection of the supporting structure should not exceed 1/500 of the span, and in walls with openings, 1/1000 of the span. In the study [8], tests of the wall made of ceramic brick 3.11 m long, 0.98 m high, and 0.1 m wide were published. The wall was built on an I-section steel beam and the test consisted of loading the wall from the top using the force simulating the load from the ceiling and forcing the relocation of the steel supporting beam. The first crack appeared at the deflection of the order of 2.5 mm that is approximately 1/1200 of the wall span. The study [9] quotes tests of full walls and walls with openings made of masonry bricks on a full scale. The walls were constructed on a steel beam deflecting with the increase in loads applied through the reinforced concrete tie beam to the top edge of the wall. In order to prevent cracking of the brick wall supported on the floor or beam element, it was recommended that the requirement of not exceeding its limit deformation characterized by the shape deformation angle of the wall Θ should be fulfilled. The studies [10,11,12] indicate results of tests on ceramic partition walls on a full scale. It was concluded that they were characterized by lower floor bending strength than the previously described scaled partitions made of ceramic elements. All described results are presented in Table 1.

A commonly used method of limiting cracking of infilling masonry walls is their expansion from the upper ceiling, for example, by filling the gap with polyurethane foam. The thickness of the expansion joint depends on the calculated value of the ceiling deflection. The most common vertical connection is steel anchoring, which is strengthening the partition in the direction perpendicular to the wall surface [13]. In the studies carried out until now, few tests of destroying non-load-bearing walls in real conditions, i.e., building structures undergoing shape deformations on a full scale, were shown. Therefore, the authors of the article undertook this task.

The appearance of cracks in partition walls with deflection of supporting floors is considered in standards as exceeding the limit condition of their usability [13,14,15,16]. Regulations concerning the limits of deflections in structures constituting support under masonry walls are often available in standards concerning the design of reinforced concrete structures, and more rarely, in standards concerning masonry structures. Selected requirements for permissible floor deformations are presented in Table 2.

The American standard ACI 318-14 [17] and its earlier editions, in the situation in which non-structural elements are designed on the floor (e.g., masonry partition walls), allows deflection values not exceeding 1/480 of the effective span of the floor. On the other hand, the American standard ACI 530-08/ASCE 5-08/TMS 402-08 [18] requires that deflections of beams and lintels from constant and variable loads should be limited to 1/600 of the span, or 7.6 mm, where the lower value is decisive. In the British standard BS 5628-2:2005 [19], it is assumed that deflection of the wall supporting structure should not exceed 1/500 of the span, or 20 mm. The German standard DIN 1045-1 [20] intended for designing reinforced concrete structures limits deflections of the floor with partition walls based on it to 1/500 of the span and imposes minimum floor thicknesses. In the Belgium standard NBN B 03-003 [21], the following recommendations concerning limit deflection of the wall supporting structure are assumed: unreinforced walls with openings—l/1000, unreinforced walls without openings or reinforced walls with openings—l/500, reinforced walls—l/350, movable walls—l/250. In the EU standard EC 6 [25], it was stipulated that usability of masonry structural elements cannot be deteriorated by the behavior of other structural elements, such as deflections of floors or walls. On the other hand, the standard does not define any rules for checking these deflections or does not indicate any limit deflections of the structures on which the walls would be constructed.

The presented literature review shows differences in the results of crack resistance of masonry walls due to deflection of supports. The requirements included in the standards [17,18,19,20,21,22,23,24] regarding the impact of building structure on non-load-bearing walls provide different permissible values for ceiling slab deflections. The reference documents do not fully describe the impact of the location of door openings in relation to the length of the walls, the conditions of the slab support, and the conditions of contact between the wall and the surrounding structures on their cracking resistance [10,11,12,13,14,15,16,26,27]. Therefore, research on the behavior of partition walls on flexible supports is still necessary. The impact of these factors on the stress state and cracking of self-supporting partition walls with door openings, made of ceramic bricks, supported on a concrete slab is the subject of this article.

## 2. Materials and Methods

In order to carry out the undertaken research study, three types of tests and analyses were performed. At first, tests of the wall samples in laboratory conditions were conducted in order to obtain material data needed for conducting numerical analyses using the finite element method (FEM). After completion of the numerical analyses, tests of full-scale walls in a building structure were carried out. The order and scope of the conducted research study are pointed below.

1. Laboratory tests: Their purpose was to characterize the used materials and obtain the strength parameters of the walls in accordance with the protocol provided in the standards [28,29,30,31,32]. The scope of the research included, among others, the following mechanical parameters of masonry materials and masonry samples:
  - Test code LAB 1—mechanical parameters of the ceramic bricks and mortar;
  - LAB 2—compressive strength of the tested wall samples in the horizontal and vertical directions;
  - LAB 3—tensile strength of the tested wall samples in the horizontal and vertical directions;
  - LAB 4—shear strength of the tested wall and its internal friction coefficient.
2. Numerical analyses performed using the Finite Element Method (FEM) in the ANSYS program environment: Mechanical parameters obtained on the basis of the above-mentioned laboratory tests were used for modeling the walls. The full mechanical characteristics of the examined walls used in modeling were the reason for the application of the homogeneous isotropic material model. Numerical analyses were carried out for walls 3.07 m high, 5.75 m long, and 12 cm thick with a door opening 2.1 m high and 0.99 m wide. The following two wall models were analyzed due to the types of their connection with the building structure along their vertical edges:
  - FEM TEST 1—free connection in the horizontal and vertical direction of the wall surface;
  - FEM TEST 2—rigid (model of toothing connection with perpendicular load-bearing walls).
3. Tests of the full-scale partitions conducted on walls constructed on reinforced concrete floors in a real building: Partitions have been prepared with the use of the same materials and protocols as in laboratory measurements and FEM analysis. Two partitions with door openings in the middle of their span and the same dimensions and types of vertical connections as in FEM analysis has been tested, which are as follows:
  - BUILD 1—vertical connection of the wall with flexible steel anchors restraining relocation only in the direction transversal to its surface;
  - BUILD 2—rigid toothing connection.

### 2.1. Materials

**Figure 2:**
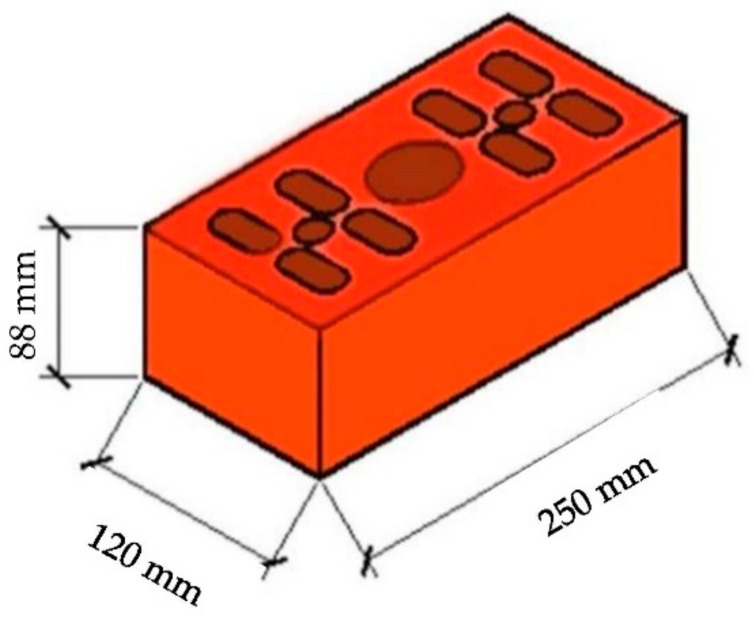
Dimensions and layout of holes for ceramic brick class M12.5 (FCP, Brest, Belarus) used in the tests.

The walls for all of the tests presented in this research: laboratory (BelNIIS laboratory, Brest, Belarus), numerical, and full scale were built using hollow (18%) ceramic brick, class M12.5 (FCP, Brest, Belarus). This is the most common ceramic brick element used for the interior partitions within the apartment in the Belarus (with a wall thickness of 12 cm). Dimensions and layout of holes for the ceramic brick are shown in Figure 2.

Tested walls have been carried out on standard concrete mortar with compressive strength of 10.9 MPa, with mortar joints 10–15 mm thick. For the preparation of masonry mortars, a factory-made dry mixture was used (FCP, Brest, Belarus).

First, the masonry elements and mortar were tested (LAB 1). The compressive strength of the mortar at the time of testing was established in accordance with the requirements of EN 1015-11 [25].

**Figure 3:**
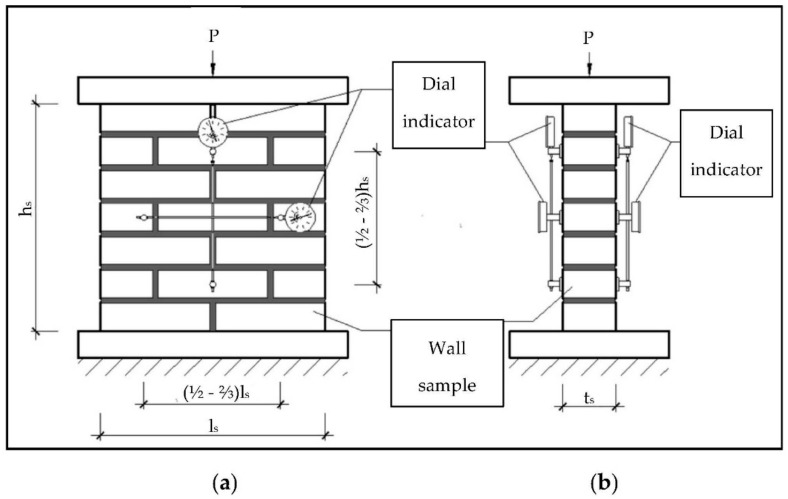
Compressive strength test in the direction perpendicular to the horizontal mortar joints of the wall samples: (**a**) front view and (**b**) side view.

Wall samples have been prepared in the laboratory and tested on the stand of own production, with the use of a 3000 kN hydraulic press (Pneumat P3000, Minsk, Belarus). Dial gauges (Baker V2, Pune, India, 0.01 mm) were used to measure the displacements of the tested samples. The production of the masonry samples, their curing, testing, and processing of test results were carried out in accordance with the standard EN 1052-1 [26,27,28]. The specimens were tested with an axial compressive load acting perpendicularly and parallel to the direction of horizontal mortar joints (LAB 2). In Figure 3, the diagram of the test stand of the compressive strength in axial load is directed perpendicularly to the horizontal mortar joints of the wall.

**Figure 4:**
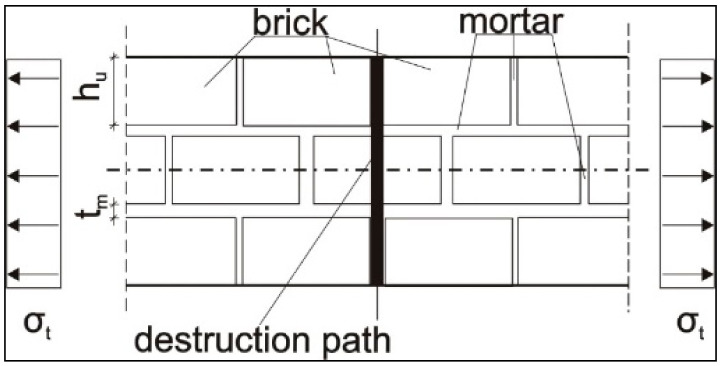
Destruction of the masonry wall sample exposed to axial tension with cracking along the joints and masonry elements.

Wall samples were also tested for tensile strength in the direction along and across to the horizontal mortar joints (LAB 3). In the case when the destruction occurs due to the rupture of the masonry element (Figure 4), the tensile strength of the wall sample is determined by Equation (1) as follows:

*f_t_*_1_ = 0.5*·f_bt_* ⋅ 1/(1 + *t_m_*/*h_u_*), (1)

where *f_bt_* is the tensile strength of the masonry element, *t_m_* is the thickness of mortar joints, and *h_u_* is the height of the masonry element.

**Figure 5:**
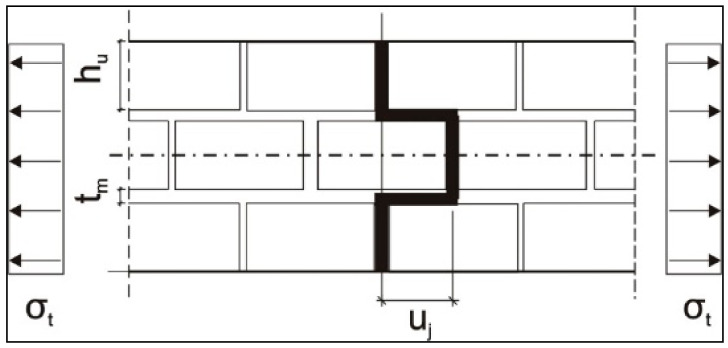
Destruction of the masonry wall sample exposed to axial tension with cracking along only the joints.

If the destruction passes along the mortar joints only, the tensile strength of the wall sample is set according to Equation (2) as follows:

*f*_*t*2_ = *f*_*v*0_·*u_j_*/(*h_u_* + *t_m_*), (2)

where *f_v_*_0_ is the initial shear strength of the masonry in the plane of the horizontal mortar joints (tangential bond), which is determined in the laboratory conditions and *u_j_* is the distance between the crack in joints and in masonry elements, according to Figure 5.

The minimal value obtained from the tests calculations is taken as a characteristic strength of the masonry under axial tension.

**Figure 6:**
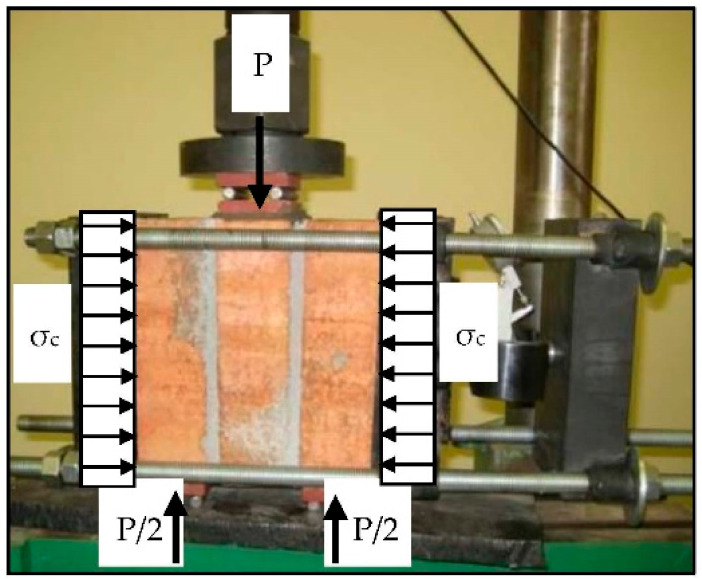
Testing of the initial shear strength and the internal friction coefficient of the masonry wall.

Initial shear strength of the masonry (tangential adhesion *f_v_*_0_) and the internal friction coefficient (*tg α*) have been established on the basis of the thawing test of masonry samples exposed to the simultaneous action of compressing and shear stresses, according to EN 1052-3 [29] (LAB 4), which is illustrated on Figure 6.

Since the magnitude of the compressive stresses during the tests was variable, it was possible to test the dependency between shear forces *f_v,i_* and compressive forces *f_c,i_*. The strength of clean-cut (tangential adhesion) *f_v_*_0_ was established by extrapolating the graph to the ordinate *f_c,i_* = 0.

### 2.2. Numerical Calculations

Numerical calculations were performed with the assumption of fully completed expansion joints from the floor on top edges of the walls, for loads from self-weight of partition walls. Calculations were conducted using the finite element method (FEM) in the computational environment of ANSYS program (AES, Canonsburg, PA, USA). The decision of using ANSYS environment for numerical analysis results from its extensive computing environment with the structural analysis module, wide library of finite elements, and experience of the authors. For the purposes of the analyses presented in this study, numerical tests of models were carried out until the serviceability limit state was exceeded. Due to the size of the analyzed problem and the comparative analyses for structures in natural conditions carried out in the next phase of the research, global FEM analyses were performed. The ceramic wall was modeled as a homogenous, isotropic material. It was possible to use macromodeling because of the wide range of wall sample tests that allowed for the implementation of mechanical properties. Macromodel results had been confirmed by the results of experimental tests. Choosing the linear flexible model was accepted for the description of material properties. The accepted criterion for crack formation is that the main tensile stresses exceed the tensile strength of the masonry in the corresponding direction.

The finite elements (FE) were implemented from the available ANSYS program library. The choice of FE was caused by different properties of the modeled elements of the structure (frame and filling wall). Pillars and beams of the frame were modeled using dual-node BEAM3 elements, with three degrees of freedom in each node (in the directions of the horizontal and vertical axis and rotation). The walls subject to the analyses were modeled using quadruple-node PLANE182 elements with two degrees of freedom in each node (translations in horizontal and vertical nodal directions) predefined for the surface elements as walls. The elements were defined with respect to coordinates, wall thickness, and elastic properties of the material. The dimensions of the wall FE are 50 mm × 50 mm. In the corners of the door opening and in the zone of contact with the adjacent construction, FE has been thickened up to the dimension of 10 mm × 10 mm. Division of the meshes has been made with FE densification within the zone of expected cracking of the material.

During the calculations, the problem of non-linear contact between the wall and elements of the building structure frame was solved. The non-linearity of contact between elements results from variable adhesion and friction of contacting surfaces in the deformation conditions. In the accepted computational models, the contact between the walls and the adjacent structures was simulated using the surface-to-surface contact finite elements (CFE). Contacting structures were considered as deformable bodies, the contacting surfaces of which form a contact pair. The target surface (frame elements) was modeled by FE TARGE169, and the contact surface (filling) was modeled by FE CONTA171. The calculations of friction used the basic Coulomb–Mohr model. In this model, two contacting surfaces can have shear stresses of a certain magnitude due to their interaction before the slip phase. This condition is known as sticking. The Coulomb– Mohr friction model determines the equivalent shear stress *τ*, in which the sliding on the surface first represents a part of the contact pressure (3) as follows:

τ = µ·p + c,(3)

where *μ* is the coefficient of friction, *p* is contact pressure, and *c* is tangential adhesion. The *μ* and *c* values are a property of the contact surface material. As soon as the limit shear stress is exceeded, the two contact surfaces slide against one another. This state is known as sliding. The grip–slip calculation determines when a point changes from grip to slip and vice versa.

### 2.3. Testing of Full-Scale Walls

**Figure 7:**
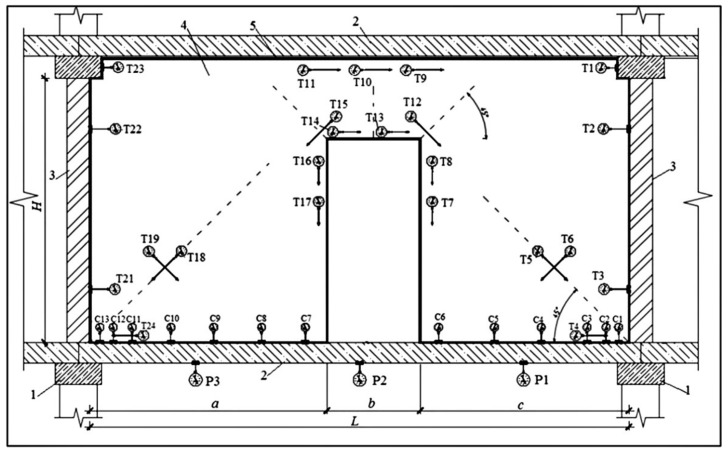
General scheme of the tested partition walls with positioned units for measurements of deformations and relocations: 1—reinforced concrete spandrel beam, 2—multi-channel ceiling slabs made of reinforced concrete, 3—masonry cross walls, 4—partition wall under test, and 5—horizontal expansion joint (T—dial deformation gauges, accuracy to 0.001 mm, C—dial relocation gauges, accuracy to 0.01 mm, P—floor deflection gauges, accuracy to 0.01 mm).

Experimental in situ tests were conducted on full-scale non-load-bearing partition walls in a framed structure residential building under construction. Two masonry walls were tested (height H = 3.07 m, length L = 5.75 m, and thickness 12 cm) with a door opening (2.1 m high and b = 0.99 m wide) in the middle of their span. The strips of walls between the opening and ceiling were reinforced at the bottom using three steel bars (diameter 12 mm) anchored in horizontal joints along the lengths of 250 mm. This type of reinforcing the wall strip above the door opening is a typical solution in the region of Minsk (Belarus) and some also appear in Poland. An expansion joint (30 mm thick) was made between the ceiling and the top edge of the wall. This type of horizontal connection between the infilling wall and the ceiling allows us to dilate these elements. It is the most commonly used connection solution in multi-story buildings in Central and Eastern Europe, studied in [10,13]. The walls were built on the floor made of prefabricated reinforced concrete multi-channel slabs (FCP, type: 240 × 90 × 24 cm^3^, Brest, Belarus) supported on reinforced concrete spandrel beams of the building frame. One of the walls was connected with cross walls using flexible steel anchors that restrained relocation only in the direction transversal to its surface (BUILD 1). The second wall was rigidly connected with the brick load-bearing cross walls by constructing toothings with it (BUILD 2). The scheme of the measurement station is shown in Figure 7.

**Figure 8:**
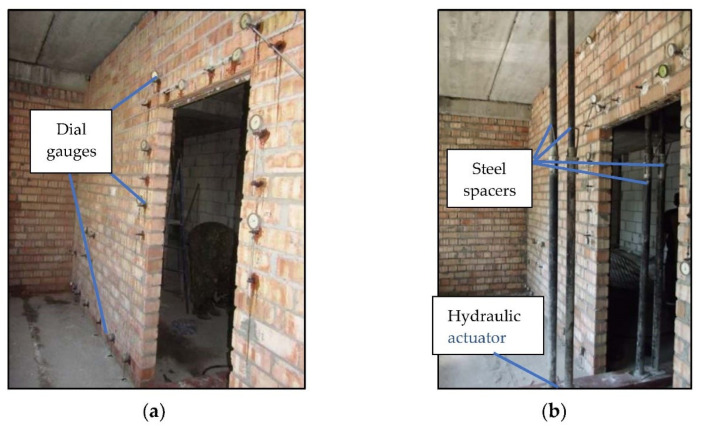
View of the partition walls under study (**a**) with fitted measuring tools and (**b**) steel spacers for floor loading.

Figure 8 presents photos of the testing stand after positioning the measuring tools. During testing of the partition wall, deflection of the floor was forced by loading it in the middle of the span using hydraulic actuators, lifting capacity 100 kN (Pneumat P100, Minsk, Belarus). In order to transfer the load to the floor under the wall, steel threaded spacers were placed between the actuators and ceilings (Figure 8b).

The floor was loaded gradually with an increase of deflection at each stage by 1 mm up to the value at which cracking of the partition wall occurred. At each loading stage, the floor deflections, vertical relocations of the bottom edge of the partition walls, and thickness of the gap appearing between them were measured. The thickness of the gap between the wall and the floor was measured using the set of steel feeler gauges (Kafer M2/20 T, Villingen-Schwenningen, Germany) with an accuracy of 0.1 mm. This allowed the determination of the length of contact between the wall and the floor. In addition, using the mechanical dial gauges (Kafer FM1000/5 T, Villingen-Schwenningen, Germany), deformation of the wall was measured with the accuracy of 0.001 mm in the direction of the trajectory of the main tensile stresses as the most dangerous as far as cracks of the wall are concerned. The location of the gauges was established based on numerical calculations of the walls under test (Figure 7 and Figure 8). The size of the measurement base depended on the direction of deformation measurement and included at least a wall element with masonry joints. Measurement readings were taken immediately after the achievement of a specific floor deflection level and after exposure of the load, within 15–20 min.

## 3. Results and Discussion

### 3.1. Results of Laboratory Measurements

Results of laboratory tests (LAB 1) on ceramic wall elements, which are the objects of further analyses are shown in Table 3.

The compressive strength of the mortar at the time of testing was established in accordance with the requirements of EN 1015-11, reaching the value of 3.1 MPa. Results of determining the strength and deformation characteristics of masonry walls (LAB 2–LAB 4) under compression along and across horizontal mortar joints have been presented in Table 4.

Results of laboratory tests concerning tensile and friction properties of the analyzed brick walls are shown in Table 5.

Presented values of mechanical properties for the wall samples were used for numerical calculations shown in Section 3.2.

### 3.2. Results of Numerical Calculations

**Figure 9:**
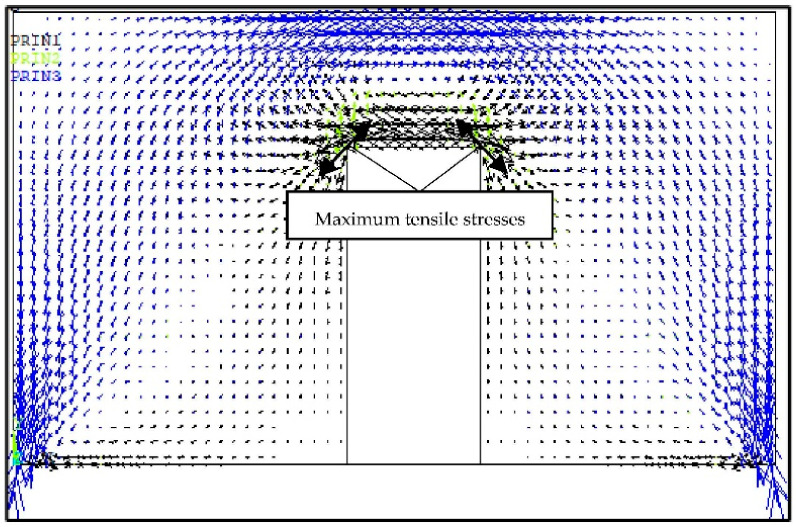
Trajectories of the main stresses in the non-load-bearing partition wall with door opening during deflection of the floor.

During the first stage of the analyses (FEM TEST 1), the calculations were performed for the walls, the vertical edges of which were not connected with vertical load-bearing structures of the building, with a door opening in the middle of their span. In Central and Eastern Europe, the most popular method of connecting non-load-bearing masonry walls along vertical edges is the use of flexible steel anchors, restraining relocation only in the direction transversal to its surface. The tests were performed in a flat stress state. For this reason, in the FEM TEST 1 calculations for this type of connection, the model free of connection in the horizontal and vertical directions in the plane of the wall surface has been used. It was determined that during deflection of the floor, the main maximum tensile stresses were concentrated in top corners of the openings at an angle of approximately 45° to the supporting joint (Figure 9).

After exceeding of admissible limit stress, shape deformations of the wall may lead both to diagonal and horizontal cracks of the wall in this zone. According to the conducted analysis, it was found as expected, that morphology of these cracks depends, among other things, on the following factors:

- the distance of door opening from the vertical edge of the wall;
- the ratio of its length to its height;
- wall strength and deformability;
- method of connecting the wall with vertical load-bearing structures of the building.

The presented factors are applicable to all technologies of masonry infill walls.

In the bottom zones of the wall, tensile stresses also appear, which have much lower values than in corners of the opening, however. The presence of the opening leads to the reduction of the main tensile stresses *σ*_1_, occurring in the zones of contact with the floor compared to the wall without opening. Values of maximum tensile stresses σ_1_ in the zone of contact between the wall and the floor constitute approximately 5–6% of the maximum value of contact compressive stresses *σ_c_* in this zone.

**Figure 10:**
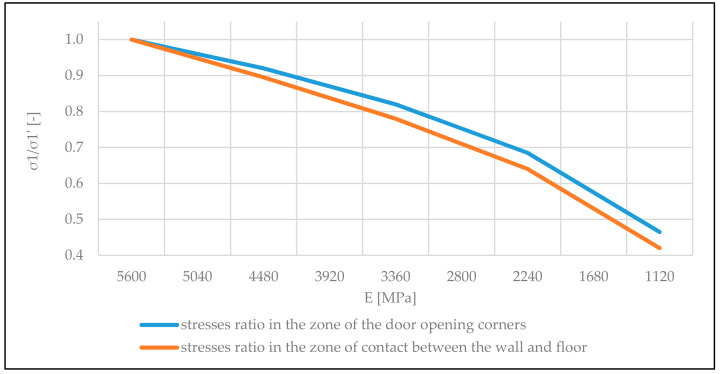
Graph of the dependency between *σ*_1_*/σ*_1*′*_ ratio and wall modulus of elasticity *E* [MPa] (*σ*_1_—main tensile stress with a lowered modulus of elasticity and *σ*_1*′*_ –stress for modulus of elasticity *E* = 5600 MPa).

As expected, on the basis of the conducted numerical tests, it was determined also that the level of wall stresses caused by deflection of the floor to a considerable extent depends on the elasticity properties of the wall. This results from the cooperation of the walls with the floor as a statically undefined structure system in which materials of the walls and floors have different deformability features, both with respect to temporary and long-term loads. The lower the flexural modulus E, the lower the values of tensile stresses in corners of the opening and in the zone of contact between the wall and the floor. The achieved dependency between the stresses in the door opening zone and flexural modulus E of the wall is presented in Figure 10.

From these regularities, it can be concluded that the stress level in non-load-bearing walls can be decreased by using the masonry elements with lower flexural modulus. It can be also stated that in walls with joints of regular thickness 10–12 mm, reduction of stresses is possible by using mortar characterized by high flexibility and deformability.

**Figure 11:**
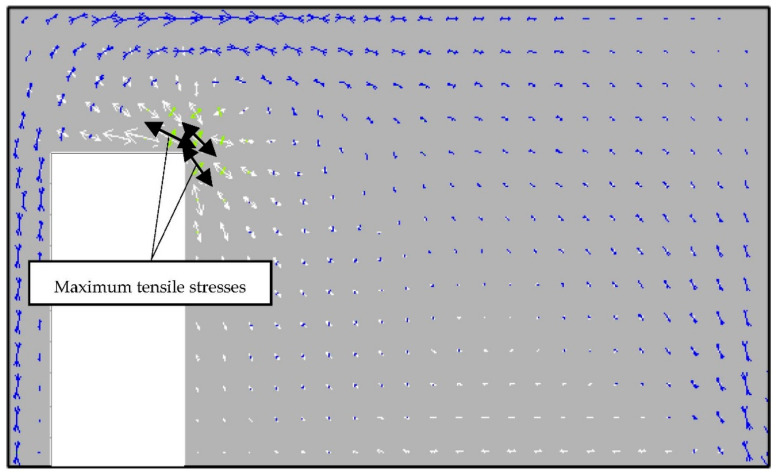
Trajectories of the main stresses in the wall with door opening moved to the vertical edge of the wall.

In the wall in which the opening is moved to one of the vertical edges, the maximum values of tensile stresses σ_1_ are located near the corner adjacent to the longer section of the wall (Figure 11).

**Figure 12:**
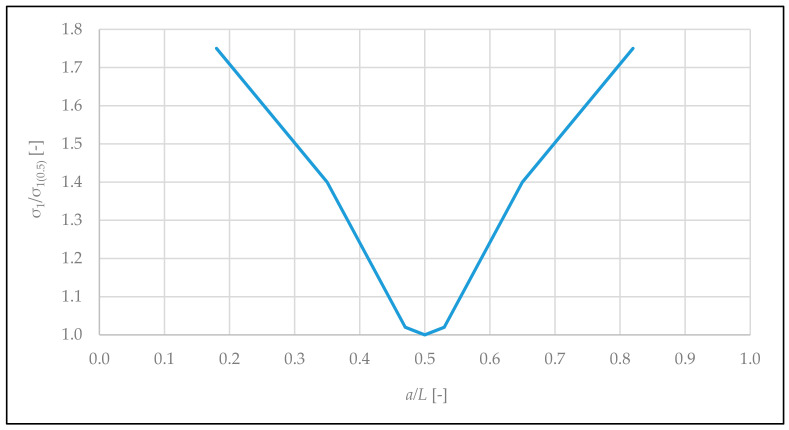
Graph of the dependency between *σ*_1_/*σ*_1 (0.5)_ ratio and door opening localization on the length of the wall. *σ*_1 (0.5)_—main tensile stresses in the zone of the door opening corners, situated in the middle part of the wall (*a*/*L* = 0.5), *a*—distance from the vertical edge of the wall to the opening center, and *L—*length of the wall.

The closer the opening is to the wall edge, the higher the values of the main tensile stresses in the zone of the corner on the longer side of the wall. Figure 12 shows the curve of maximum values of *σ*_1_ in the corner versus the location of the door opening.

**Figure 13:**
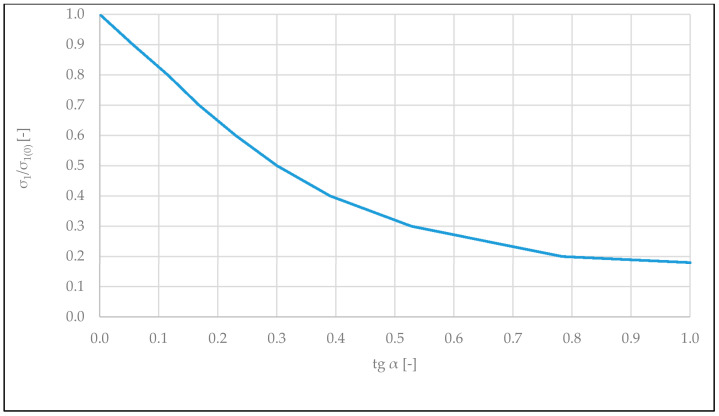
Graph of *σ*_1*/*_*σ*_1 (0)_*—tg α* dependency (*σ*_1(0)_—the main tensile stresses in the zone of door corner at the coefficient of friction tg α equal to 0).

The level of the main tensile stresses *σ*_1_ in the corners of the opening in walls not connected along horizontal edges with the load-bearing structure of the building is considerably affected by the value of the coefficient of friction (*tg α*) between the masonry wall and floor. Figure 13 presents the dependence *σ*_1_/*σ*_1(0)_*—tg α*, where *σ*_1(0)_ are the main tensile stresses in the zone of door corners at *tg α* = 0.

With the increase of friction coefficient *tg α* from 0 (no friction) to 1 (rigid connection with the floor), stresses *σ*_1_ in the zone of the door opening corners decrease more than five times. On the other hand, tangential stresses operating in the zone of contact between the wall and floor increase. Exceeding *f_v_*_0*,obs*_ (shear strength stresses of the wall along supporting joints) may lead to horizontal shearing of the wall in the zone of contact with the floor. Prevention of this is possible by transferring spreading forces *T* directly to load-bearing structural elements of the building along vertical joints of the wall.

According to the completed numerical tests, in the case of the rigid connection of the wall with vertical load-bearing structures of the building (FEM TEST 2), with the increase of the floor deflection, the main tensile stresses increase only in the zone of contact between the wall and floor. In the door opening corners, the values of σ_1_ are insignificant and practically do not depend on the floor deflection level. In addition, a comparative analysis of the main stresses in walls with the door opening and full walls was conducted. It was concluded that with equal values of floor deflection, the main tensile stresses in the zone of contact with the floor in the wall with door opening were approximately 25% lower than in the wall without opening. This results from higher flexural rigidity of the wall without opening and thus its lower ability to adapt to deflection of the floor.

### 3.3. Experimental Tests in the Facility

**Figure 14:**
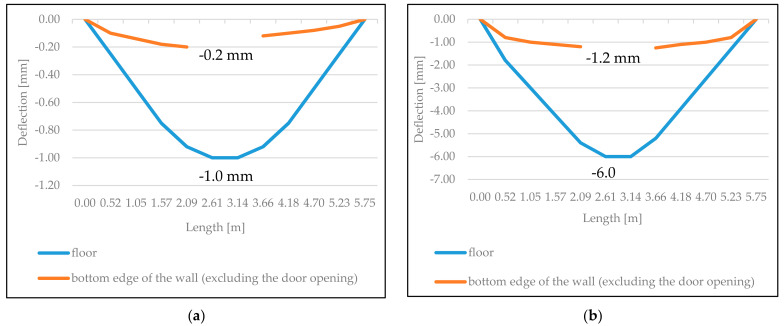
Graphs of deflection of the floor and bottom edge of the partition wall not connected with cross walls. (**a**) Wall at the first stage of loading and (**b**) wall at the last stage of loading.

Figure 14 presents the graphs of deflection for reinforced concrete floor and vertical relocations of the bottom edge of the partition wall not connected with cross walls at the first and last stage of loading (BUILD 1).

**Figure 15:**
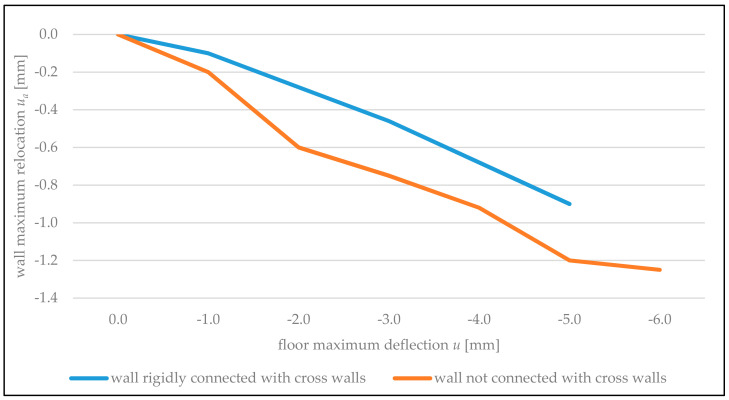
Graphs of maximum vertical relocations of the bottom edge of the wall (*u_a_*), depending on maximum deflection of the floor (*u*).

As it can be concluded from the graphs, as early as the first stage of the floor loading, a gap appears between the floor and bottom edge of the walls, and the thickness of this gap increases with the increase of the floor deflection. This is illustrated by dependencies between maximum vertical relocations of the bottom edge of the walls *u_a_* (at the end of the edge of the vertical door openings) and deflection of the floor *u* in the middle of its span shown in Figure 15. During the final stage of loading, the maximum deflection of the floor exceeded maximum vertical relocations of the bottom edge of the walls by approximately five times. Vertical relocations of the bottom edge of the partition wall stiffened using cross walls (BUILD 2) were 1.5–2 times lower than for the wall not connected with cross walls. The comparison of experimental and theoretical vertical relocations of bottom edges of the walls indicates that with deflection of the floor in the middle of its span *u =* 1–5 mm, the discrepancy in the values of these relocations did not exceed 15%.

**Figure 16:**
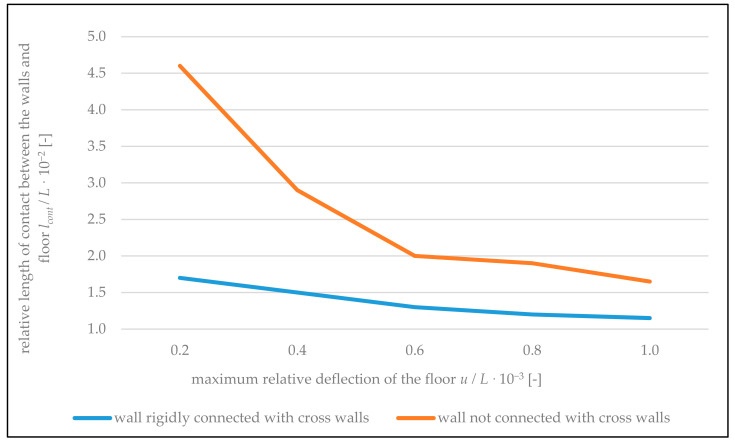
Graphs of the relative length of contact between the walls and floor *l_cont/_L*, depending on maximum relative deflection of the floor *u*/*L*.

Distortion of the contact between the floor and bottom edge of the wall starts at the very beginning of the floor bending. As a result of the loosening of the wall from the floor, with the increase of its deflections, the length of contact between the wall and floor decreases. For example, with the floor deflection in the middle of its span of *u* = 1.0 mm, the length of its contact with the wall not connected with cross walls was 28 cm, and with the deflection of *u =* 6.1 mm, the length of contact decreased down to the value of 9.3 cm. In the case of the wall rigidly connected with cross walls with the floor deflection of *u =* 1.2 mm, the length of contact between the floor and wall was approximately 10 cm, and with the deflection of *u =* 5.3 mm, this length was 8 cm. Graphs of the relative length of the zone of contact between the wall and floor (*l_cont_/L*) depending on relative deflection *u*/*L* of the floor are shown in Figure 16.

Any change of the length of contact between the partition wall and floor causes redistribution of contact stresses. Their concentration occurs in the zones with minimum floor deflections that are in supporting zones. In these zones, the wall is loaded with contact stresses causing its local pressing perpendicularly to supporting joints. This can lead to local crushing of the wall or the occurrence of diagonal cracks in corner areas of the partition walls. One positive effect of this redistribution is the reduction of bending moments in the floor from loading caused by partition walls. The local nature of load transfer from partition walls to floors with distortion of contact between them is not always included in standard provisions. For instance, according to standard [33], 60% of the self-weight of the partition wall with door opening are transferred to the floor as a uniform linear load along the wall length. On the other hand, the remaining 40% are transferred to the floor in the form of concentrated forces applied on the section at 1/3 length of the wall from floor supports to the door opening. In European standards, the load from partition walls is usually assumed to be linear or surface. These recommendations are contradictory to the presented results from the completed tests.

**Figure 17:**
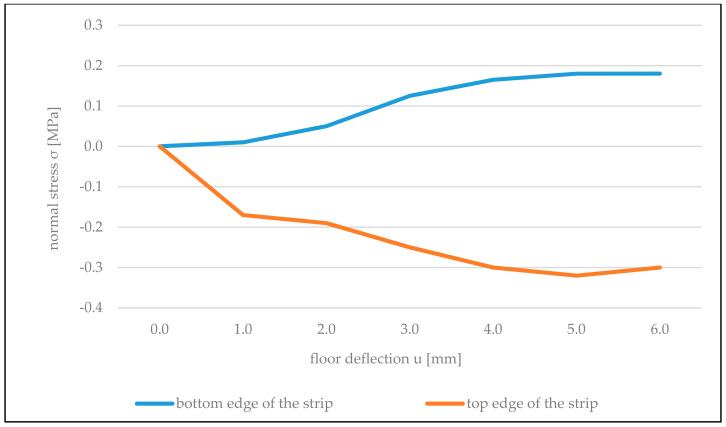
Graphs of changes in normal stresses on edges of the wall strip above the opening in the wall not connected with cross walls, depending on the floor deflection *u*.

Figure 17 illustrates the dependencies of normal stresses operating in the direction parallel to the supporting joints on the bottom and top edge of the strip of wall above the opening on the floor deflection value *u.* These stresses are defined as a product of Young’s modulus *E* and wall deformations *ε* obtained based on measurements using gauges T13 and T10 (Figure 6).

Compression of the top edge of the strip and expansion of the bottom one indicates the eccentric load on the strip in the direction of its length, which was caused by balanced spreading forces *T* occurring on the contact of the wall with the floor. Values of tensile stresses on the bottom edge of the strip at maximum floor deflections are similar to the tensile strength of the wall along supporting joints *f_t,cal_* = 0.22 MPa (Table 3).

**Figure 18:**
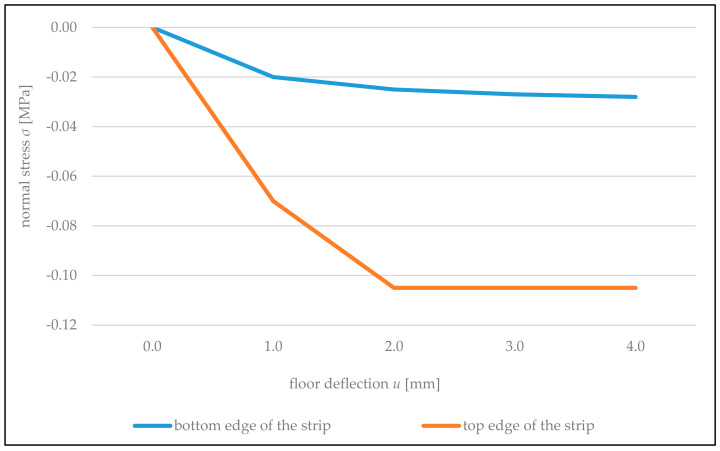
Graphs of changes in regular stresses on edges of the wall strip above the opening in the wall rigidly connected with cross walls, depending on the floor deflection *u*.

In the case of rigid connection of the partition wall with cross walls, the wall strip above the opening also acted as eccentrically compressed (Figure 18). The entire cross section of the strip was compressed, and the value of maximum compressing stresses was approximately three times lower than in the case of the partition wall not connected with cross walls (Figure 17). It is connected with the reduction of horizontal deformations of the wall by vertical structural elements of the building.

**Figure 19:**
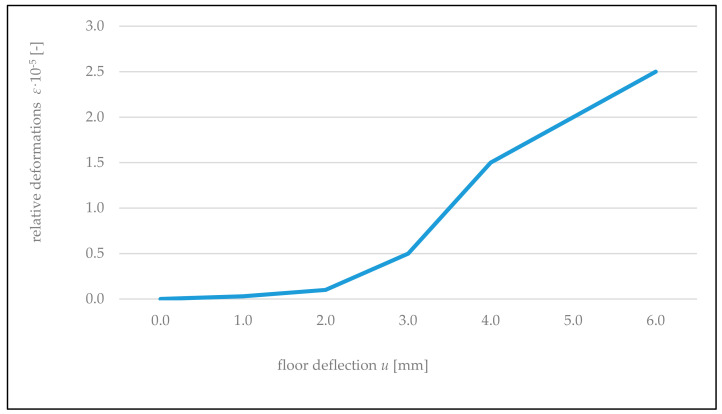
The dependency of floor deflection *u* on the relative deformations ε of the wall at an angle of 45° in corners of the door opening for the wall not connected with cross walls.

It was established also that near the contact with the floor, expansion of the wall along supporting joints occurred. In the wall rigidly connected along vertical edges, the values of expansion deformations were 1.5–2 times lower than in the wall connected freely. In both cases, stresses caused by these deformations did not exceed the tensile strength of the wall along supporting joints (*f_t,cal_ =* 0.22 MPa, according to Table 1). In the wall not connected with cross walls, using gauges T7, T8, T16, and T17 (Figure 6), expansion deformations of the wall were recorded along vertical edges of the opening in the direction perpendicular to supporting joints. Values of these deformations were increasing when the door opening corners were approached. The most strained areas of the wall are corners of the opening where maximum tensile stresses operate at an angle of 45° to vertical supporting joints. Dependencies of relative wall deformations ε in this direction recorded using gauges T12 and T15 (Figure 6) on the wall deflections *u* are shown in Figure 19. In the case of the wall connected with cross walls, the wall contributed to compression both along vertical edges of the door opening and at an angle of 45° to supporting joints in the zone of its corners. This results from stiffening of the partition wall with load-bearing cross walls because in top areas of the partition wall, according to measurements using gauges T1, T2, T22, and T23 (Figure 6), the wall contributed to expansion along supporting joints, while in bottom areas, according to gauges T3 and T21, it contributed to compression.

**Figure 20:**
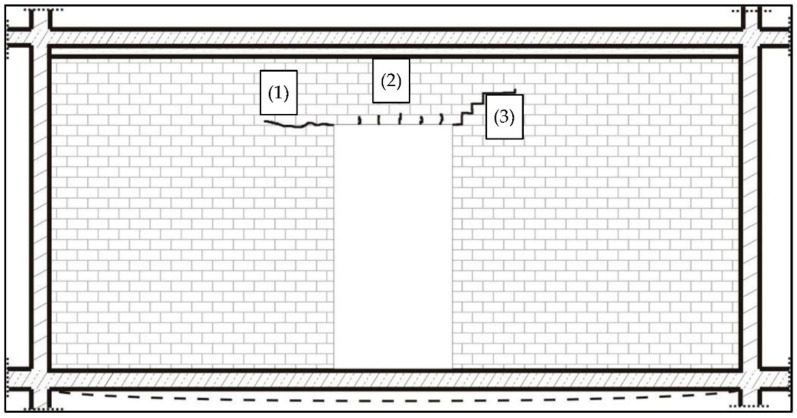
Character and sequence of wall cracks on the wall not connected with cross walls; (**1**)—horizontal crack; (**2**)—vertical crack; and (**3**)—oblique crack.

Damage of the partition wall not connected with cross walls took place as a result of cracking of the wall in the zone of the door opening corners. The wall cracks occurred in the form and sequence shown in Figure 20.

Horizontal crack (1) appeared in the supporting join, vertical cracks (2) appeared in vertical joints, and oblique crack (3) had a shape of stairs matching vertical and supporting joints. The cracks appeared suddenly, with deflection of the floor in the middle of its span at the value above 6 mm, that is, relative deflection of *u*/*L* = 1/958. Crack (2) appeared with tensile stresses on the bottom edge of the wall strip above the opening *σ =* 0.18 MPa, close to the tensile strength of the wall along supporting joints *f_t,cal_* *=* 0.22 MPa (Table 3). Crack (3) appeared at maximum values of the wall deformation of *ε* = 2.5 × 10^−5^, that is, stress *σ* = 0.14 MPa comparable with the tensile strength of the wall across supporting joints *f_w,obs_* = 0.16 MPa (Table 5).

As mentioned already, in the wall rigidly connected with cross walls (BUILD 2), compressive stresses occurred in the area of the opening corners and in the wall strip above the opening (Figure 16) at all stages of the floor loading. It had a significant effect on the crack resistance of the wall. At the maximum achieved deflection of the floor u = 12 mm, that is, at the relative deflection of *u*/*L* = 12/5750 = 1/479, no cracks or damage was noticed in the examined wall.

### 3.4. Research Findings and Their Application

On the basis of the FEM TEST 1 numerical analyses, the relationships between the wall flexural modulus and tensile stresses in the area of the door opening were derived. It has been confirmed that the use of masonry elements or a mortar with a lower elasticity coefficient leads to a reduction in the tensile stress value in the corner zone of the door opening. The derived dependencies allow for the calculation of theoretical stresses for the given elasticity of the designed partition walls.

The relationship between friction coefficient *tg α* (between the tested wall and foundation) and the maximum tensile stresses in the area of the door opening has been obtained. It has been calculated that with the increase of friction coefficient *tg α* from 0 (no friction) to 1 (rigid connection with the floor), stresses in the zone of the door opening corners decrease more than five times.

The relationship between the location of the door opening along the wall length and the value of the maximum tensile stresses in the area above the door opening was derived. It has been shown that the location of the door opening close to the edge of the wall leads to an increase in the tensile stress value by 175% compared to the location of the door opening in the middle of the wall length.

The conducted analyses may have practical application for the calculation of tensile stresses in non-load-bearing walls made of brick elements with a door opening and in the design of this type of structure.

Numerical analysis of the wall with rigid connection with vertical structure (FEM TEST 2) indicated that with the increase of the floor deflection, the main tensile stresses increase only in the zone of contact between the wall and floor. In the door opening corners, the values of σ_1_ are insignificant and practically do not depend on the floor deflection level.

On the basis of the experimental tests, it was found that the value of the relative deflection of the foundation *u*/*L*, which led to the cracking of the wall with the door opening in the middle of the span, freely connected along the vertical edges (BUILD 1), was 1/958. The BUILD 1 experiment (carried out on a natural scale in a real building) showed that the deformation of the foundation that is causing cracking of the non-load-bearing masonry wall was comparable with the results of wall tests on the 1:3 scale carried out by Pfeffermann [7]. The rigid connection of the vertical edges of the wall with the building structure performed in the BUILD 2 experiment allowed us to avoid cracking in the extent of the relative foundation deflection *u*/*L* of 1/479. This value allows the avoidance of cracking of walls erected on foundations with a maximum deflection in the range provided by all standards referred to in Table 2 [17,18,19,20,21,22,23,24]. Relying on these results, the authors would suggest to project and perform the masonry partitions with rigid connection along their vertical edges.

In the future, it is planned to carry out measurements and experiments on the walls with different masonry elements, different static schemes of the foundation, and varied patterns of openings in the walls.

Results of the presented research are limited to the analyzed type of materials and connections of the walls with the structure of the building.

## 4. Conclusions

The performed tests and analyses allowed for the conclusions presented below.

- The most strained area of masonry partition walls supported on reinforced concrete floors are corners of door openings together with the strip of wall above them;
- Cracking of the wall in this area occurs mainly as a result of tensile stresses, which appeared, in the case of the lack of rigid connection, between vertical edges of the wall with adjacent vertical load-bearing structures;
- The appearance of a gap between the bottom edge of the wall and the floor results in redistribution of load favorable for the floor from the weight of the partition wall, which concentrates in supporting zones of the floor;
- In the case of the completed experimental tests, walls made of ceramic brick with the span of 6 m and vertical edges connected freely with the building structure cracked at the relative deflection of the floor under the wall, with the value equal to 1/958 of the floor’s length;
- Deformation of the foundation equal to 1/958 that is causing cracking of the tested wall BUILD 1 was comparable with the results of wall tests on the 1: 3 scale carried out by Pfeffermann [7];
- Application of rigid connection along vertical edges of the walls, while maintaining appropriate expansion joints along both horizontal edges, allowed the avoidance of cracking of the wall across the entire measuring range. The wall was not damaged at the floor deflection equal to 1/479 of its length;
- Floor deflection of 1/479 does not exceed the limit deflection values provided in the standards;
- The measured tensile strength of the tested walls in the horizontal direction was 0.22 MPa;
- Strength value obtained in laboratory tests of the wall samples corresponded to destructive values measured for the full-scale wall built in the construction site;
- The numerical analyses allowed the determination of dependency between the change in location of the door opening along the length of the tested walls and tensile stresses in the zone of its corners;
- It was concluded that the value of the main tensile stresses in the corners of the opening in walls not connected along vertical edges with the load-bearing structure of the building depends on the friction coefficient (tg α) between the masonry wall and the deflecting floor. With the increase of friction coefficient tgα from value 0 (free connection) to 1 (rigid connection in the horizontal direction), the values of tensile stresses in the zone of the door opening corner decrease by five times;
- The conducted analyses may have practical application for the calculation of tensile stresses in non-load-bearing walls made of brick elements with a door opening and in the design of this type of structure.

## Figures and Tables

**Table 1:** Critical deflections and stresses in the state of cracking of brick partition walls.

Ordinal Number	Author and Date	Wall Type and Description	Bending Tensile Strength	Ratio of Deflection of the Supporting Structure to Its Span at Cracking
1	Meyerhof G., 1953 [4]	External brick walls without openings; different dimensions; analytical approach	0.21	1/2000
2	Beranek 1983 [5]	External brick walls with openings; different dimensions; analytical, and experimental approach	0.1–0.3	1/2000
3	Rolanda et al., 2003 [6]	Brick wall without opening in a scale of 1:3, experimental approach	-	1/1000
4	Pfeffermann et al. 1981 [7];	Brick wall with an opening in a scale of 1:3, experimental approach	-	1/946
5	Loots et al.l 2004 [8]	Brick wall without opening in a scale of 1:2.5, experimental approach	-	1/1200
6	Piekarczyk 2019 [9]	Brick walls with (a) and without opening (b) asymmetrically loaded, experimental laboratory approach	-	1/1700 (a)1/2800 (b)

**Table 2:** Comparison of permissible deflection values of the supporting structure under partition walls.

Ordinal Number	Standard No	Country/Region	Maximal Ratio of Deflection of the Supporting Structure to Its Span or Maximal Deflection	Additional Information
			[-] or [mm]	
1	ACI 318-08 [17]	USA	1/480	-
2	ACI-530-08/ASCE 5-08/TMS 402-08 [18]	USA	1/600 or 7.6 mm	the lower value is decisive
3	BS 5628-2 [19]	UK	1/500 or 20 mm	the lower value is decisive
4	DIN 1045-1 [20]	Germany	1/500	-
5	NBN B 03-003 [21]	Belgium	1/1000 or 1/500 *	*—valid for unreinforced walls with openings
6	EN 13747: 2005 [22]	European Union	1/350 or1/500 *	*—valid for brick walls
7	EN 1992-1-1: 2008 [23]	European Union	1/250 or 1/500 *	*—value valid for the deflections affecting the non-load-bearing walls
8	PN-B-03264: 2002 [24]	Poland		

**Table 3:** Average values of strength characteristics of ceramic bricks with a voidness V = 18%.

Average Normalized Strength according to EN 772-1 ^1^	Compressive Strength in the Direction Horizontal to the Stretcher Surface	Flexural Tensile Strength	Shear Strength	Axial Tensile Strength
*f_by,mv_* (MPa)	*f_bx,mv_* (MPa)	*f_btb,mv_* (MPa)	*f_bv,mv_* (MPa)	*f_bt,mv_* (MPa)
18.37	7.50	2.97	2.81	0.99

^1^ In the direction perpendicular to the stretcher surface.

**Table 4:** Characteristic values of strength and deformation properties of the tested wall samples under compression along horizontal (*x*) and vertical (*y*) directions.

Compressive Strength of Masonry Walls (MPa)		$\frac{f_{cy,mv}}{f_{cx,mv}}$	Short-Term Modulus of Elasticity E (MPa)		$\frac{E_{y,mv}}{E_{x,mv}}$	Lateral Deformation Coefficient	
*f_cy,mv_*	*f_cx,mv_*		*E_y,mv_*	*E_x,mv_*		*ν_xy,mv_*	*ν_yx,mv_*
5.2	3.5	1.49	5400	5642	0.96	0.26	0.27

**Table 5:** Characteristic values of mechanical properties for the tested partition walls.

Initial Shear Strength	Internal Friction Coefficient	Tensile Strength across Supporting Joints	Tensile Strength along Supporting Joints
*f_v_*_0*,obs*_ (MPa)	*Tg α* (-)	*f_w,obs_* (MPa)	*f_t,cal_* (MPa)
0.18	0.63	0.16	0.22
